# Assessment of Early Breast Cancer Response to Chemotherapy with Ultrasound Radiomics

**DOI:** 10.3390/diagnostics16060948

**Published:** 2026-03-23

**Authors:** Swapnil Dolui, Basak Dogan, Corinne Wessner, Jessica Porembka, Priscilla Machado, Bersu Ozcan, Nisha Unni, Maysa Abu Khalaf, Flemming Forsberg, Kibo Nam, Kenneth Hoyt

**Affiliations:** 1 Department of Biomedical Engineering, Texas A&M University, College Station, TX 77845, USA; swapnil.dolui@tamu.edu; 2 Department of Radiology, University of Texas Southwestern Medical Center, Dallas, TX 75390, USA; 3 Department of Radiology, Thomas Jefferson University, Philadelphia, PA 19107, USAflemming.forsberg@jefferson.edu (F.F.);; 4 Department of Internal Medicine, University of Texas Southwestern Medical Center, Dallas, TX 75390, USA; nisha.unni@utsouthwestern.edu; 5 Department of Medical Oncology, Thomas Jefferson University, Philadelphia, PA 19107, USA; 6 Department of Small Animal Clinical Sciences, Texas A&M University, College Station, TX 77845, USA; 7 Department of Electrical and Computer Engineering, Texas A&M University, College Station, TX 77845, USA

**Keywords:** breast cancer, multiparametric analysis, neoadjuvant chemotherapy, radiomics, ultrasound imaging

## Abstract

**Objective**: This prospective study investigated the use of H-scan ultrasound (US) imaging as a novel component of a multiparametric radiomic analysis framework for characterizing human breast cancer response to neoadjuvant chemotherapy (NAC) before and early after treatment initiation. **Methods**: Thirty breast cancer patients scheduled for NAC were scanned using a clinical US system (Logiq E9, GE HealthCare) equipped with a 9L-D linear array transducer. Radiofrequency (RF) data was obtained at baseline (pre-NAC) and after 10% and 30% of the complete dose of chemotherapy. The RF data was analyzed by a bank of 256 frequency-shifted bandpass filters to form H-scan US frequency images. Grayscale texture features were extracted from both B-scan and H-scan US images. In addition, US attenuation coefficient and speckle statistics based on the Nakagami and Burr distributions were estimated from the RF data. Data classification of tumor and peri-tumoral regions was performed using a novel three-dimensional (3D) score map based on support vector machine (SVM) modeling. Unlike conventional classifiers that report only a single prediction score, a 3D score map provides a visual representation of the classifier decision space, enabling interpretation of class separation and treatment-induced shifts in multiparametric US measurements. **Results**: The dataset was split into 10 disjoint partitions (90% training, 10% testing) to compute area under the receiver operating characteristic curve (AUC), sensitivity, specificity, and accuracy measures. Actual patient response to NAC was assessed at surgery and categorized as either pathologic complete response (pCR) or non-pCR. Multiparametric US and data classification results at pre-NAC found AUC values of 0.78 after using only tumor information (*p* < 0.01), which increased to 0.81 with inclusion of peri-tumoral information (*p* < 0.01). Significant differences in multiparametric US measures from both cancer response types was found after integration of patient data collected at 10% completion of the NAC regimen (i.e., first NAC cycle), yielding an improved AUC of 0.86 (*p* < 0.001). **Conclusions**: Multiparametric US imaging with radiomic features from both the tumor and peri-tumoral regions is a promising noninvasive approach for monitoring early breast cancer response to NAC.

## 1. Introduction

Breast cancer accounts for nearly 30% of all new cancer diagnoses in women. Over the last three decades, breast cancer mortality has reduced considerably [1]. Despite the encouraging clinical advances that have spurred this change, breast cancer remains a major public health issue with major economic impact. Preoperative neoadjuvant chemotherapy (NAC) is a standard of care for locally advanced breast cancer patients, which facilitates tumor size reduction and can make inoperable tumors resectable. If a tumor is responsive, NAC can reduce the risks of the surgical strategy, allowing the tumors to be removed with a lumpectomy or partial mastectomy rather than a more complicated complete mastectomy [2]. Beyond surgical downstaging, NAC provides several additional clinical advantages. It allows for the in vivo assessment of tumor chemosensitivity, enabling early identification of treatment-responsive versus resistant disease. Pathologic complete response (pCR) after NAC has been shown to correlate with improved long-term outcomes, particularly in triple-negative and HER2-positive breast cancer and serves as an important surrogate endpoint in clinical trials. NAC also facilitates axillary downstaging, potentially reducing the need for axillary lymph node dissection and its associated morbidity. Importantly, the neoadjuvant setting offers a unique opportunity to tailor subsequent adjuvant therapy based on residual disease burden, thereby individualizing treatment escalation or de-escalation strategies [3,4,5].

Pathologic evaluation of residual disease following surgery, including residual cancer burden (RCB) scoring, is currently the reference standard for assessing NAC efficacy, but it is inherently retrospective and cannot inform real-time treatment adaptation [6]. Consequently, there remains a critical unmet need for robust, standardized imaging biomarkers capable of accurately predicting treatment response during NAC and enabling response-adaptive therapy. Although several imaging modalities are routinely used to monitor response to NAC, there is no single standardized imaging biomarker that reliably and universally predicts pathologic response or long-term outcome across breast cancer subtypes. In clinical practice, response assessment is most commonly based on serial anatomic measurements of tumor size using conventional imaging, primarily contrast-enhanced breast magnetic resonance imaging (MRI), supplemented by mammography and ultrasound (US), following Response Evaluation Criteria in Solid Tumors (RECIST)-based or similar criteria [7,8,9]. Among these, breast MRI has demonstrated the highest accuracy for estimating residual tumor burden, particularly in triple-negative and HER2-positive disease, though its performance is more limited in hormone receptor-positive cancers and in the presence of treatment-related fibrosis [10,11]. However, noticeable changes in tumor size are often absent at the interim timepoints, which limits early tumor response assessment and may incur unnecessary patient toxicity and a significant cost burden. Specialized MRI techniques like diffusion-weighted MRI and dynamic contrast-enhanced MRI have also been explored for use in monitoring early NAC response in breast cancer patients [12,13]. However, MRI is not always available and lengthy exam times can produce patient discomfort and claustrophobia [14]. As an alternative, US can be used as a cost and time-efficient imaging modality for breast cancer evaluation. The use of contrast-enhanced US and spectral mammography has shown some promise for NAC monitoring [15,16]. However, studies in large patient populations are needed to validate these initial findings [17]. Empirical data suggest that qualitative US imaging yielded performance comparable to that of MRI in detecting early NAC effects [18]. Further technological advancements are necessary to enhance the efficacy of US imaging in monitoring NAC. Recent studies exploring US data using deep learning-based approaches have enabled automated data-driven modeling of imaging patterns and improved predictive performance [19]. These methods typically require large datasets to achieve robust generalization for specific cancer subtypes and may be sensitive to variations in data acquisition settings [20,21]. Therefore, traditional radiomics frameworks based on well-defined mathematical descriptors, combined with interpretable machine learning models, remain valuable, particularly in studies with limited cohort sizes [22].

Speckle statistics in US describes the mathematical properties of the characteristic granular pattern seen in US images of soft tissues [23]. These statistics yield quantitative metrics that help characterize the underlying tissue structures. The data is typically represented using a probability density function (PDF) to model the distribution of the envelope of backscattered US signals. This approach allows for a more precise and objective assessment of tissue properties based on the speckle patterns observed in US images. Known as the Nakagami distribution, this classical model assumes the presence of many random identical US scatterers within tissue [24]. A more recent alternative assumes that multiscale sub-resolved US scatterers in tissue follow a power law distribution. This leads to a Burr probability distribution of speckle amplitudes [25]. The main difference in the Nakagami and Burr distribution is present in the exponential versus power law tails, respectively. Beyond speckle statistics, several other quantitative US imaging techniques based on the spectral properties of the backscattered radiofrequency (RF) data have been developed for tissue characterization. For example, US attenuation coefficients can correlate with structural changes and can help distinguish malignant from healthy breast tissues [26]. In a large cohort of breast cancer patients, quantitative US imaging using a collection of different parametric measurements was able to predict tumor response to NAC within 8 weeks after start-of-treatment [27].

Recently, a newer US modality to investigate the backscattering properties of cell aggregates has emerged. Termed H-scan, this imaging approach is based on classification theory for US scattering in soft tissue [28]. This uses a series of frequency-shifted Gaussian filters applied to backscattered RF signals. Matched filter outputs are used to provide local discrimination between various-sized US scatterers or tissue structures [29]. In general, lower frequency spectral content is generated from larger scattering structures, whereas higher frequency signal content is produced by a US wave interacting with an ensemble of smaller scatterers on a scale below the wavelength of the US transmit pulse. H-scan US imaging uses a simple mathematical framework and avoids complex calibration for tissue characterization. In addition, the use of short convolutional filters makes H-scan US frequency imaging well-suited for real-time implementation. The H-scan US frequency images depict relative scatterer size and may capture chemotherapy-induced structural changes within the tumor microenvironment [30,31,32]. This clinical study proposes H-scan US frequency imaging and texture analysis as a novel component of a multiparametric radiomic analysis for the early prediction of breast cancer response to NAC.

Texture analysis has been widely used to quantitatively describe the spatial relationship of neighboring pixel intensity or localized spectral elements within US images [33]. Texture analysis usually relies on data described by a gray-level (GL) co-occurrence matrix (GLCM), run length matrix (GLRLM), size zone matrix (GLSZM), or a neighborhood gray-tone difference matrix (NGTDM) [34,35]. GLCM extracts pixel-pair relationships in a localized region. Techniques such as GLRLM and GLSZM investigate pixel connectivity as a direction-dependent and -independent measure respectively. NGTDM assesses the difference in neighborhood pixels centering around the one under consideration within a localized region [35]. These matrices collectively highlight the distribution pixel intensity patterns which are then summarized into various quantitative statistical measures of image texture such as homogeneity, morphology, and periodicity. There are numerous radiomic features and distinct approaches to characterize and classify different image texture patterns that have been explored. Texture analysis is combined with quantitative US to provide additional insight into tissue organization patterns and NAC treatment-induced alterations in breast cancer [36,37].

Using data from an ongoing multisite clinical study involving patients newly diagnosed with breast cancer and scheduled to receive NAC, we present findings on the use of a multiparametric US radiomic approach that combines H-scan US frequency, B-scan US, speckle statistics, attenuation coefficient values, and texture analysis with machine learning classification techniques for predicting breast cancer response to NAC. In this study we also propose a three-dimensional (3D) score map that provides feedback and visualization of the multiparametric US data across NAC timepoints, which may help clinicians assess the trajectory of an early cancer response.

## 2. Materials and Methods

### 2.1. Patient Selection and Treatment

This study involved Thomas Jefferson University (Philadelphia, PA) and the University of Texas Southwestern Medical Center (Dallas, TX). The clinical protocol (NCT04715958) was approved by the institutional review board at both sites and the Food and Drug Administration (FDA) under an Investigational New Drug application (IND 112241). Patients with a biopsy-confirmed diagnosis of breast cancer without distant metastasis and who decided to be treated with NAC were considered eligible for the study. Patients were enrolled after obtaining written consent. Exclusion criteria for the study included breast-feeding, on-going pregnancy, allergies to chemotherapy agents or similar compounds, and any severe medical or psychiatric comorbidities. Human epidermal growth factor receptor-2 (HER2), estrogen receptor (ER), progesterone receptor (PR), and Ki-67 status were obtained from breast biopsy pathologic reports as standard of care. Ki-67 levels were categorized as low (score < 15%), average (score of 16 to 30%), or high (score > 31%), and used to assess tumor proliferation [38]. The treating oncologist determined the specific chemotherapy regimen for each patient, which consisted mostly of anthracycline and taxane-based drugs. Following completion of 4 to 6 months of NAC, patients underwent surgery for removal of any residual tumor tissue. Tumor response was classified either as a pathologic complete responder (pCR) or non-pCR based on the surgical specimen. RCB scores were obtained from post-surgical pathological evaluation following completion of NAC and were extracted from the corresponding clinical records.

### 2.2. Data Acquisition

Patients underwent US imaging before the start of NAC (pre-treatment) and again after 10% and 30% completion of the NAC regimen. US examinations were performed using a Logiq E9 system (GE HealthCare, Waukesha, WI, USA) equipped with a 9L-D linear array transducer (bandwidth 3 to 8 MHz). US imaging was performed by an experienced sonographer or radiologist with a single focus placed at the center of the tumor location. RF data was acquired from both sagittal and transversal planes and analyzed offline using custom software (MATLAB 2024a, MathWorks Inc., Natick, MA, USA). Tumor dimensions were measured at baseline (pre-NAC) and after the end of NAC (typically 4 to 6 months later). Tumors depicted in the B-scan US images were identified and lesion boundaries were de-lineated by an experienced radiologist blinded to study results or patient outcome. These segmentation boundaries were then enlarged by 3 to 10 mm from the tumor boundary to evaluate the use of surrounding tissues during patient analysis.

### 2.3. B-Scan US Imaging

Given the backscattered RF signal r(z), where z is the axial distance (depth), and including the backscattered coefficient of tissue and combined beam effects of the transducer, the US signal $\hat{r}\left(z\right)$ can be described as:

(1) $$\hat{r}\left(z\right)=F^{-1}[e^{2\alpha fz}R(f,z)]TGC(z)$$

where $R\left(f,z\right)$ is the Fourier transform (F) of $r\left(z\right)$, superscript “−1” denotes inverse operator, α is the attenuation coefficient (units Np/cm/MHz), f is frequency (units MHz), and TGC(z) is a time gain compensation operator that amplifies the received US signal as a function of distance z (units cm). The time gain compensation is a frequency-independent variable, so at depth $z,\;\;TGC\left(z\right)$ is treated as a constant quantity [39]. The amplitude or envelope a(z) of the backscattered US signal for each scan line can be extracted as follows:

(2) $$a\left(z\right)=\left|\hat{r}\left(z\right)+H\left[\hat{r}\left(z\right)\right]\right|$$

where H is the Hilbert operator and $\left|\cdot \right|$ denotes absolute value. Typical B-scan US images are formed after applying logarithmic compression to US signal envelope.

### 2.4. US Speckle Statistics

US speckle is the deterministic, noise-like pattern that appears in US images due to constructive and destructive interference of scattered US waves. Probabilistic modeling of speckle patterns provides a description of tissue architecture. The Nakagami distribution can be used to describe tissue scattering from similar-sized sources [40]. After normalization by the data size, the histogram of US signal amplitudes described by Equation (2) can be expressed as a two-parameter PDF:

(3) $$p_{Nag}(a,m,Ω)=\frac{2m^{m}a^{2m-1}}{\Gamma \left(m\right){Ω}^{m}}exp\left(-\frac{m}{Ω}a^{2}\right)\;$$

where Γ is the gamma function, m and Ω are shape and scale parameters, respectively. However, US scattering by sub-resolved multiscale-sized structures is better characterized by the following two-parameter density function known as the Burr distribution [11]:

(4) $$p_{Burr}(a,b,\lambda )=\frac{2a\left(b-1\right)}{\lambda^{2}{\left[{\left(\frac{a}{\lambda }\right)}^{2}+1\right]}^{b}}$$

where b and λ are the shape and scale parameters, respectively. Both the Nakagami and Burr distributions are controlled by two different shape (m, b) and scale (Ω, λ) parameters that are primarily influenced by the range and magnitude of the speckle (envelope) amplitude data, respectively. To capture local differences in scattering behavior across the tumor region, speckle statistics were computed from the envelope of the backscattered US signal to enable localized characterization of the underlying tissue microstructure. Speckle maps were then generated to estimate shape and scale parameters using a sliding spatial window (109 × 9 samples or 1.5 × 1.5 mm) with 98% overlap along both axes.

### 2.5. US Attenuation Coefficient Measurement

**Figure 1:**
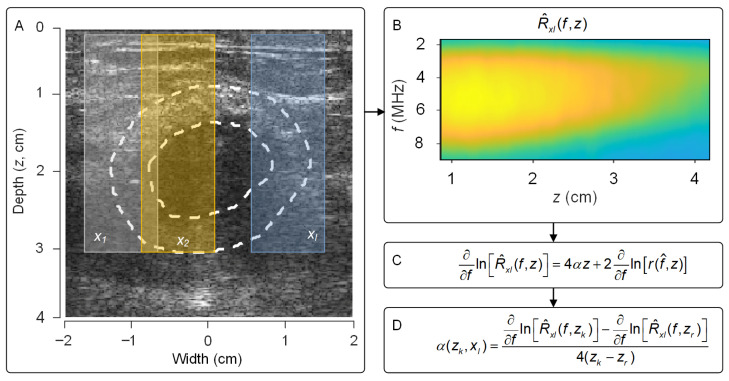
(**A**) Tumor and peri-tumor tissue regions (dotted lines) enclosed within multiple lateral estimation subregions $x_{l}$. (**B**) The localized spectral profile ${\hat{R}}_{xl}(f,z)$ was assessed from each estimation region. (**C**) The change in the $ln[{\hat{R}}_{xl}(f,z)]$ along the frequency f-axis was calculated and (**D**) attenuation coefficients $\alpha \left(f,z=z_{k},\;x\;=\;x_{l}\right)$ were estimated by applying a normalized first-order derivative to the $ln\left[{\hat{R}}_{xl}(f,z=z_{k})\right]$ term with respect to the same at reference depths $z=z_{k}$, r ≠ k. Any estimations outside the peri-tumor tissue region were eliminated from the analysis.

A US attenuation coefficient estimation map was constructed using the reference frequency-based technique [39]. In this approach, the entire pre-focal region including the peri-tumor region was considered. During implementation, the area was split into overlapping estimation regions $x_{l}$ (kernel width = 10 mm) along the lateral direction with a step size of 0.2 mm (Figure 1A). Within each of these estimation regions, the localized frequency-domain profile of $\hat{R}\left(f,z\right)$ was assessed using a sliding window (3 mm) with 95% overlap along the axial direction and averaged laterally (Figure 1B). The localized average spectral profile of $\hat{r}\left(z\right)$ at estimation region $x_{l}$ can be described as:

(5) $${\hat{R}}_{x_{l}}\left(f,z\right)={\left|e^{2\alpha fz}\;R(f,z)\right|}^{2}\;TGC{\left(z\right)}^{2}$$

A first-order differentiation was then performed on the natural logarithm of Equation (5) to eliminate the impact of the depth-dependent TGC system variable:

(6) $$\frac{\partial }{\partial f}ln\;\left[{\hat{R}}_{xl}(f,z)\right]=4\alpha z+2\frac{\partial }{\partial f}ln\;\left[R(f,z)\right]$$

Considering ${\hat{R}}_{xl}(f,z)$, the effect of the US backscattered coefficient on tissue attenuation estimation was minimized by normalizing the $\frac{\partial }{\partial f}ln\;\left[{\hat{R}}_{xl}(f,z=z_{k})\right]\;$ term with respect to a first-order differentiation from multiple reference depths $z_{r}$. Normalization of Equation (6) and rearranging terms yields the following equation:

(7) $$\alpha \left(z_{k}{,x}_{l}\right)=\frac{1}{4(z_{k}-z_{r})}\left(\frac{\partial }{\partial f}ln\;\left[{\hat{R}}_{xl}(f,z_{k})\right]-\frac{\partial }{\partial f}ln\;\left[{\hat{R}}_{xl}\left(f,z_{r}\right)\right]\;\right)\;for\;r\neq k$$

For each spatial location ($z_{k},x_{l}$), a US attenuation coefficient α was estimated by solving Equation (7) using a least mean square fit with boundary constraints defined within a −2 to 0 dB/cm/MHz range [41]. Note that any estimate outside the peri-tumor boundary was rejected from the analysis. For this study, US attenuation coefficient estimation in the pre-focal region was validated using a calibrated phantom material (Model 040GSE, Sun Nuclear Corp, Melbourne, FL, USA) prior to analysis [42].

### 2.6. H-Scan US Frequency Imaging

**Figure 2:**
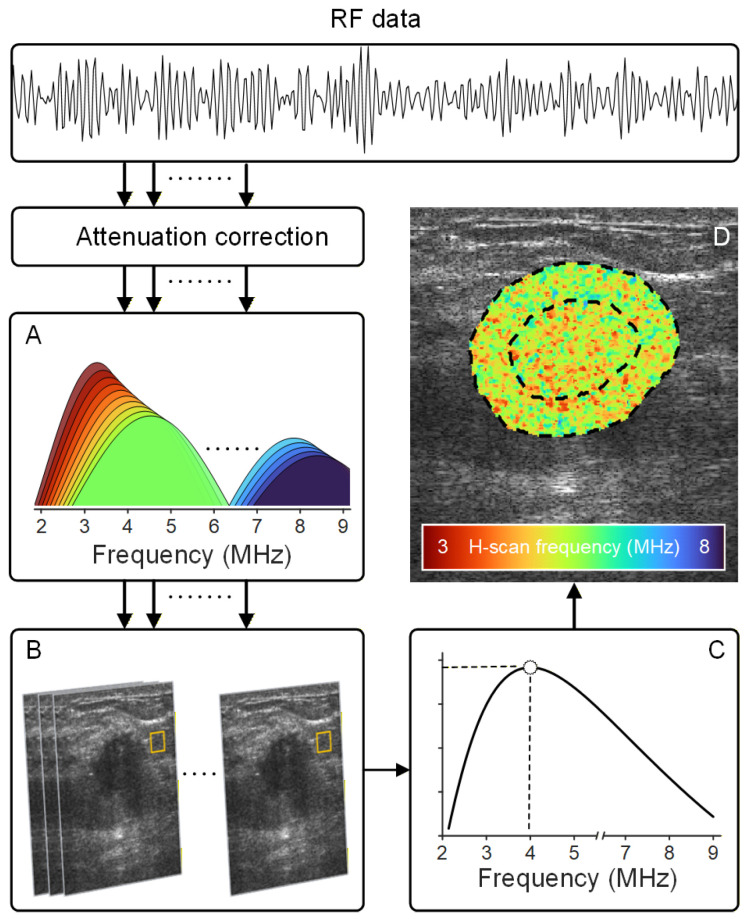
After attenuation correction, radiofrequency (RF) data was processed using a set of (**A**) 256 frequency-shifted Gaussian filters that span the entire ultrasound (US) transducer bandwidth. (**B**) At each spatial (pixel, yellow box) location, (**C**) maximum filter outputs were determined and (**D**) color-coded to form the final H-scan frequency image that describes relative US scatterer size. US measurements were obtained from the tumor and peri-tumoral regions (dotted lines).

H-scan US frequency is a novel imaging technique that reveals the spectral content associated with microstructural changes in tissue. The H-scan US analysis is a matched filter approach for the extraction of frequency information related to local changes in US scatterer size [43]. Attenuation correction was performed on RF signal prior to H-scan US image analysis [44]. In this study, spectral analysis was performed using 256 different frequency-shifted Gaussian filters with center frequencies $f_{c}$ distributed across the 3 to 8 MHz range [45]. Uniformly spaced 0.02 MHz apart, each filter had a bandwidth of $0.5f_{c}$ (relative bandwidth of 50%) (Figure 2A). Attenuation-corrected US signals were convolved with these filters to extract frequency-dependent features for further analysis. The advantage of using a variable bandwidth matched filtering approach is the preservation of spatial features. The H-scan US frequency analysis at each spatial location had a corresponding set of 256 convolution values from which a maximum was selected according to the concept of a matched filter, and it was color-coded to form the final H-scan US image (Figure 2C,D).

### 2.7. Radiomic Feature Extraction

B-scan and H-scan US image-derived texture (radiomic) features are calculated using statistical relationships described by specific data structures like the gray-level co-occurrence matrix (GLCM), gray-level run-length matrix (GLRLM), gray-level size zone matrix (GLSZM), and neighborhood gray-tone difference matrix (NGTDM) [35]. In this study, 40 texture parameters were extracted from each US image after isotropic resampling. The GLCM and GLRLM assess the distribution of pixel pairs and individual pixels, respectively, along four different angulated directions (i.e., 0, 45, 135, and 270°) before averaging. While averaging, the texture matrices were weighted based on the neighborhood pixel distances. Similarly, other techniques like GLSZM and NGTDM also assess the localized pixel relationship independent of any direction. While GLSZM investigates the connectivity of pixels, NGTDM looks at the variation of pixels within a neighborhood. Different texture parameters derived from the various matrix structures include: (1) GLCM-based contrast (CON), correlation (COR), homogeneity (HOM), and variance (VAR); (2) GLRLM-based long run low GL emphasis (LRLGE), short run emphasis (SRE), and run-length variance (RLV); (3) GLSZM-based small zone low GL emphasis (SZLGE), large zone low GL emphasis (LZLGE), large zone high GL emphasis (LZHGE) and zone-size variance (ZSV); and (4) NGTDM-based busyness (BUS) and coarseness (CRS) [35]. Texture parametric maps were constructed using a sliding window (9 × 9 samples or 1.5 × 1.5 mm) with 80% overlap along both axial and lateral dimensions. During computation of texture parametric maps, pixel intensities within each sliding window were quantized to 64 levels using the window-specific localized histogram. Note that B-scan and H-scan US images were denoised using an anisotropic diffusion filter prior to texture feature extraction. Image denoising was performed using spatially local operations confined to each individual image, without the use of cohort-level statistics or cross-patient information. This quantization step was also performed independently for each image to ensure that no cross-patient information was used during preprocessing.

### 2.8. Multiparametric US Analysis

**Figure 3:**
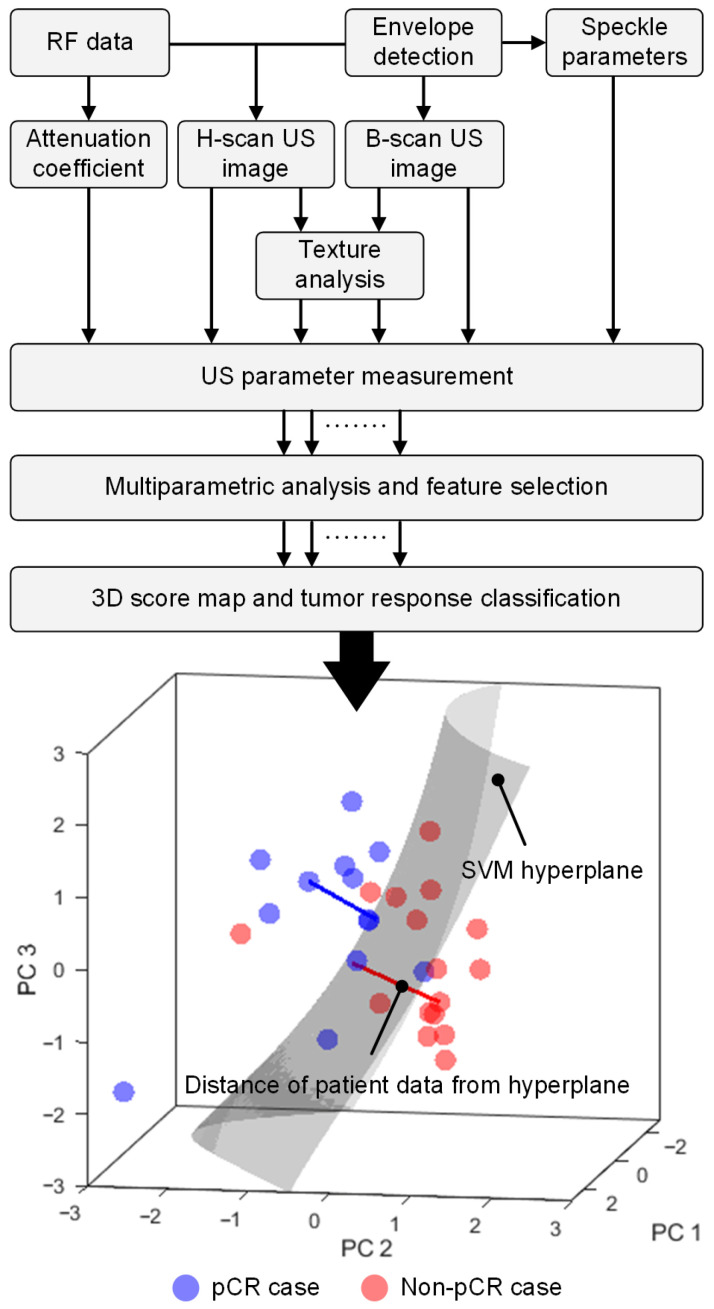
Flowchart summarizes the processing of backscattered US data (RF format) to produce different US parameter measurements. Following data reduction and use of the first three principal components (PCs), an optimal US parameter combination was formed from all features considered. A three-dimensional (3D) score map and tumor response classification was then performed using a nonlinear support vector machine (SVM) technique. The 3D score map visualizes the SVM decision hyperplane and tumor response. The signed distance of patient data from the hyperplane determines the tumor response score whereby a distance greater than 0 predicts pathologic complete response (pCR) and a distance less than 0 predicts a non-pCR to neoadjuvant chemotherapy (NAC).

A total of 174 different US or radiomic features describing both tumor and peri-tumor information from 87 US parametric maps were derived at each of the three different NAC timepoints. US parametric maps were also categorized into either time (US speckle statistics, B-scan US-derived) or frequency (US attenuation coefficient estimates and H-scan US-derived) domain-derived measures. To improve reliability and robustness of pCR detection, a multiparametric US analysis was performed to assess the separation between both groups at pre-NAC and early NAC timepoints after completion of 10 and 30% of the total chemotherapy dose. Patient-specific baseline (or pre-NAC data) values were used along with the changes in US parameters after start of NAC and relative to baseline measures. Baseline normalization was performed on a per-patient basis using each subject’s own baseline reference values rather than a group mean to prevent cross-patient information leakage. A schematic of the workflow including postprocessing steps after US data acquisition at each timepoint is presented in Figure 3.

The multiparametric US analysis was performed in 10 disjoint partitions, where 90% of the data was allocated to a training set and the remaining 10% of the data was blinded as a test cohort (*n* = 3). US parametric selection, model building, and hyperparameter tuning were performed on the training cohort. Due to the small number of patients in each test partition, evaluating classification performance per fold may introduce instability and increase the risk of optimistic bias in small cohorts. To mitigate this, for each patient only the multiparametric US scores from the held-out folds were retained, which were generated exclusively from models trained without that patient across all folds and iterations. These scores were aggregated across all folds and then averaged across iterations to obtain a single cross-validated score per patient. The resulting average scores from the entire patient cohort were subsequently used to summarize classification performance using metrics including the area under the receiver operating characteristic curve (AUC), accuracy, sensitivity, and specificity. The aggregated patient level scores were then bootstrapped to estimate confidence intervals for the performance metrics.

US parameter selection was performed in three stages within the training partition. In the first stage of US parameter selection for the multiparametric US analysis, the stability of each US parameter alone due to spatial perturbation was determined using an intraclass correlation coefficient (ICC), and those with values greater than 0.90 were retained for further analysis [46]. For early NAC timepoints, the stability of US parameters was assumed to match each patient baseline score, thereby capturing treatment-related differences among patients. In the second phase, the selected US parameters were sorted using a support vector machine (SVM) recursive feature elimination (RFE) technique [47,48]. The highest-ranked parameters were retained while the redundant features were eliminated from further consideration. In the last phase, multiple US parametric combinations were then systematically and incrementally formed by sequentially adding the sorted US parameters [49]. As shown in Figure 3, a 3D score map was used to assess the classification performance of each US parametric combination. The variance in classification performance due to the limited dataset was evaluated by repeatedly splitting the training set with *k*-fold cross-validation (CV, *k* = 3, 5, 10), which was iterated 3 times. The performance of each validation fold was stabilized by averaging across five 3D score maps generated from bootstrapped samples of its training set. In addition to finalizing US parameter selection, the performance of the 3D score map was further explored through a grid search of model hyperparameters for optimized performance of the multiparametric US analysis. Please note that ICC computation, US parameter selection, and model hyperparameter optimization were performed strictly using the training dataset of each fold. Optimized AUC scores from the nested CV, US parameters, and tuned model hyperparameters were used for the final multiparametric US analysis of the blinded test partition. Feature selection, PC coefficients, and development of the 3D score map were performed strictly within the training cohort. The multiparametric US score of the test cohort was determined using the 3D score map after filtering US parameters and applying PC coefficients derived from the training cohort.

### 2.9. 3D Score Map

A patient-specific multiparametric US score was determined from a 3D score map, which was generated by a nonlinear SVM radial basis function (RBF). The nonlinear SVM was trained using the first three principal components (denoted PC1, PC2, and PC3) of the z-score-normalized training data. As illustrated in Figure 3, a tumor response decision hyperplane was interpreted by the 3D score map. The distance between each patient data location and the SVM hyperplane was used as a metric for the multiparametric US tumor response score. In general, higher positive scores indicate that a particular patient has a higher probability of being in the pCR group and negative scores belong to the non-pCR group. Note that PC analysis was performed to reduce overfitting and model complexity to interpret the distance from the hyperplane. During training, a major problem in handling practical datasets is the imbalance of group sizes, which could cause bias in classification towards the majority group. To effectively handle the imbalance, a synthetic minority oversampling technique (SMOTE) was applied prior to training the model [50]. The performance of the 3D score map was also explored by modifying the shape of the decision hyperplane by including a grid search over model hyperparameters such as box constraint (10^−4^ to 10^0^) and kernel scale (10^0^ to 10^1^) for improved predictive accuracy. The finalized US parametric combination, z-score normalization, PC coefficients and model hyperparameters were used to retrain the 3D score map with the entire training data from the outer cohort. After training the 3D score map, the decision boundary for each training fold was recalibrated for maximizing balanced accuracy (or mean of the sensitivity and specificity values) using the multiparametric US scores derived from the corresponding SMOTE balanced training datasets. Lastly, the multiparametric US scores were then computed for the corresponding test dataset using the trained 3D score map after dimensionality reduction using the PC coefficients determined from the training set.

### 2.10. Statistical Analysis

Statistical tests were performed on multiparametric US image-based tumor response scores using either tumor or peri-tumor tissue information and compared with that obtained by combining both regions. A Shapiro–Wilk test was used to confirm data normality. A two-sample *t*-test was performed to compare the independent pCR and non-pCR groups. SVM classification performance was assessed using AUC, sensitivity, specificity, and accuracy measures. Pearson’s correlation coefficients were used to relate H-scan US frequency to baseline Ki-67 scores of cancer cell proliferation. The sensitivity of B-scan and H-scan US image intensities in the peri-tumoral region thickness from 3 to 10 mm was evaluated by Spearman’s rank correlation analysis of RCB scores. Any *p*-value less than 0.05 was considered statistically significant. All data analyses were performed using GraphPad Prism 10 software (Boston, MA, USA).

## 3. Results

### 3.1. Patient Characteristics

A total of 30 women diagnosed with invasive breast cancer and scheduled to receive NAC treatment were included in the study. Relevant patient-specific information is listed in Table 1. Of the study participants, 14 achieved pCR and the remaining 16 did not (non-pCR). Median ages of the pCR and non-pCR patients were 49 years (range 27 to 69 years) and 58 years (range 38 to 68 years), respectively. Pretreatment median tumor size for the pCR and non-pCR patient groups were both 2.5 cm with a range of 0.9 to 5.0 cm and 1.0 to 7.2 cm, respectively (*p* = 0.48). Invasive ductal carcinoma was the predominant diagnosis for most patients with 14 and 15 cases belonging to the pCR and non-pCR groups, respectively. The remaining non-pCR patient was diagnosed with invasive micropapillary carcinoma. Molecular status was determined from the initial tumor biopsy.

### 3.2. Single-Parameter US Analysis

**Figure 4:**
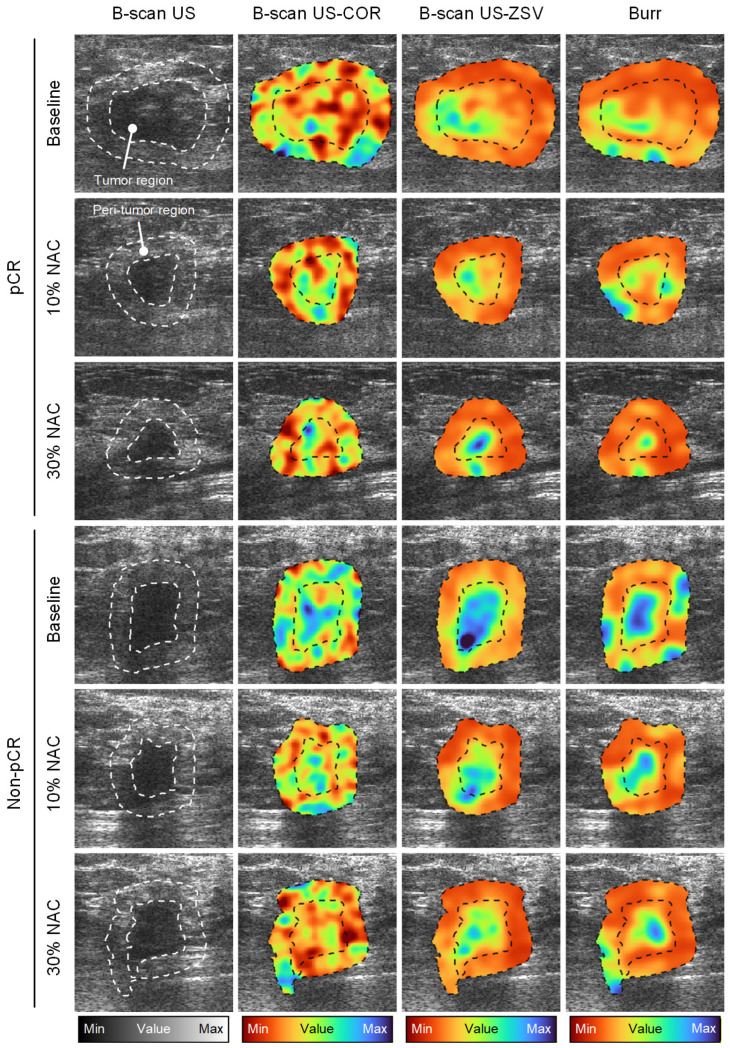
Representative amplitude-based US spatial maps at baseline (pre-NAC) and after 10 and 30% completion of a NAC regimen from patients in the pCR or non-pCR groups. B-scan US image (**first column**) and texture maps including the correlation (COR, **second column**) and zone size variance (ZSV, **third column**) features were generated from the same US images. US speckle statistics (*b* parameter from a Burr distribution) are also depicted (**fourth column**). US measurements were obtained from the tumor and peri-tumoral regions (dotted lines).

**Figure 5:**
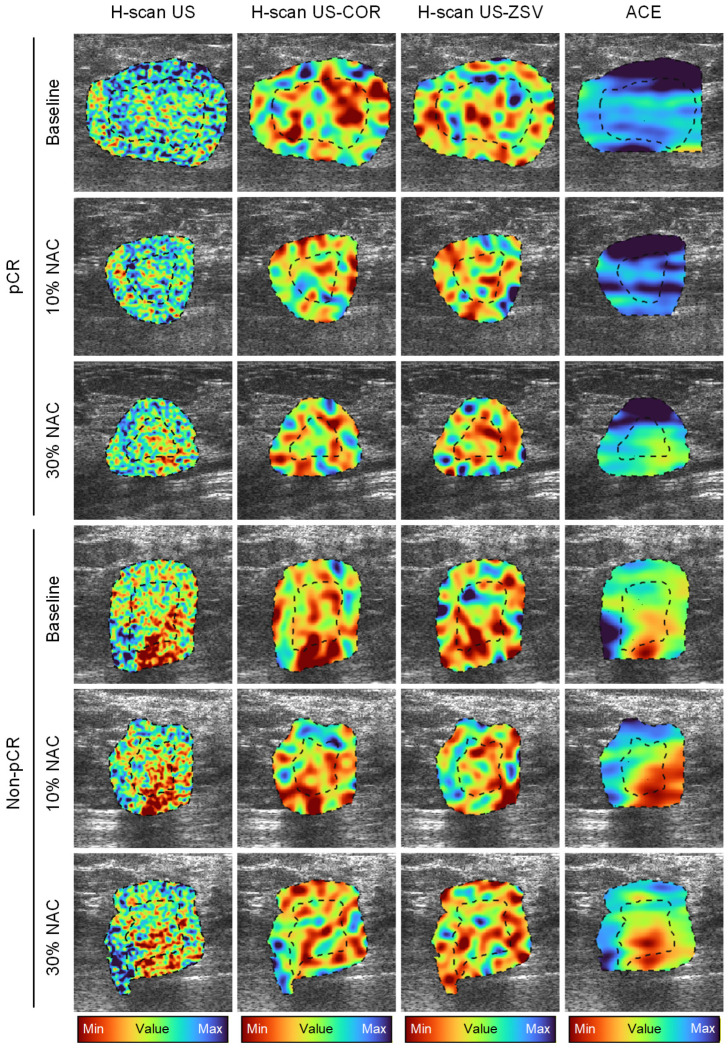
Representative frequency-based US spatial maps at baseline (pre-NAC) and after 10 and 30% completion of a NAC regimen from patients in the pCR or non-pCR groups. H-scan US image (**first column**) and texture maps including the COR (**second column**) and ZSV (**third column**) features were generated from the same US images. Attenuation coefficient estimate (ACE) is also depicted (**fourth column**).

Lesion boundaries were delineated on the B-scan US images and used for segmentation of the tumor and peri-tumoral regions. Sensitivity analysis of peri-tumoral tissue width demonstrated that a 5 mm margin yielded the strongest association with RCB scores at pre-NAC (*r* = 0.42, *p* = 0.03), compared with 3 mm (*r* = 0.41, *p* = 0.03), 7 mm (*r* = 0.40, *p* = 0.04), and 10 mm (*r* = 0.39, *p* = 0.05). While larger region widths (greater than 7 mm) showed slightly higher correlations at early treatment follow-up (10% NAC), these associations were not statistically significant (*r* < 0.35, *p* > 0.08). Notably, correlations across all surrounding tumor tissue decreased after 30% completion of NAC, which suggests reduced discriminative information (see Table 2). Considering performance across all timepoints and imaging-derived measurements, the 5 mm peri tumoral configuration provided the most balanced and stable relationship to patient RCB, supporting its selection as the primary surrounding tissue region for the multiparametric US analysis. Note that this same tumor margin width was also used in other quantitative US imaging-based clinical studies [51]. Sensitivity at follow-up timepoint is measured using relative changes in US measurements. Representative amplitude-based maps including B-scan US images before and after texture analysis (COR and ZSV) and US speckle statistic maps from a pCR and non-pCR patient before and after start of NAC are presented in Figure 4. Similarly, representative frequency-based maps including the H-scan US image with the corresponding texture analysis as well as attenuation coefficient from a pCR and non-pCR patient before and after NAC initiation are presented in Figure 5. Inspection of these results reveals differences in the H-scan US frequency images at baseline and subtle longitudinal changes after start of NAC. While analyzing texture maps derived from the B-scan US and H-scan US images, the differences in both pre-NAC and longitudinal measurements between the groups were more pronounced than the parent US images. Visible differences were also observed in frequency-based attenuation coefficient estimation maps at baseline (pre-NAC). A summary of these findings across all three timepoints is provided in Table 3. At pre-NAC, only 16 of the 176 US parameters showed statistical differences between both groups. Notably, all 16 were frequency-based measures, and more importantly, 15 of them originated from the peri-tumoral region. While evaluating the relative changes in US measurements, eight amplitude-based and five frequency-based peri-tumor US measurements demonstrated differences after 10% completion of NAC, but none were sufficient enough alone to serve as reliable tumor response predictors. More specifically, an AUC of 0.53 (95% CI = [0.41, 0.65]), 0.56 ([0.42, 0.69]) and 0.41([0.28, 0.52]) was achieved when an SVM-based classification model was trained using single US parameters at pre-NAC and after the partial completion of treatment timepoints, respectively. During the single US parameter-based classification trials, H-scan US frequency (tumor) and frequency-based BUS (peri-tumor) were most frequently selected at pre-NAC. For early NAC changes, frequency-based CON (tumor), alongside amplitude-based COR (peri-tumor) were most often selected. In addition to having prognostic value in assessing tumor response to NAC, the H-scan US frequency data had a significant correlation with baseline Ki-67 scores of cancer cell proliferation (*r* = 0.59, *p* < 0.001).

### 3.3. Multiple US Parameter Selection

**Figure 6:**
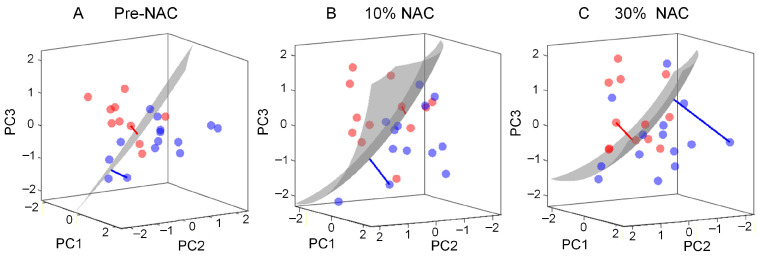
Representative 3D score map illustrating the trajectory of the multiparametric US data at (**A**) baseline and after (**B**) 10% and (**C**) 30% completion of an NAC regimen. The signed distance (red and blue lines) of groups (pCR: blue and non-pCR: red circle) increases from the decision hyperplane (grey) after including relative changes in US parameters at early time periods after start of NAC, with US parameters acquired prior to beginning of any treatment.

To improve the accuracy of tumor response prediction, a robust multiparametric US analysis for pCR prediction was performed using a 3D score map (see Figure 3). The 3D score map combines the US parameters and displays the trajectories of multiparametric US data over time following the treatment initiation. As shown in Figure 6, the multiparametric US data of both groups are separable at pre-NAC. After inclusion of relative changes in US measures after the first treatment cycle (10% treatment completion), the multiparametric US data move further from the decision hyperplane. This increase in SVM-distance after including relative changes in US measures may indicate that multiparametric US can detect treatment-induced structural changes in breast cancers. The multiparametric US score or distance between the data points and the decision hyperplane indicates the probability of the patient being in the pCR or non-pCR group. Therefore, the multiparametric approach shown in Figure 6 enables clinicians to visualize the trajectory of tumor response progression, without prior knowledge of individual US parameters.

**Figure 7:**
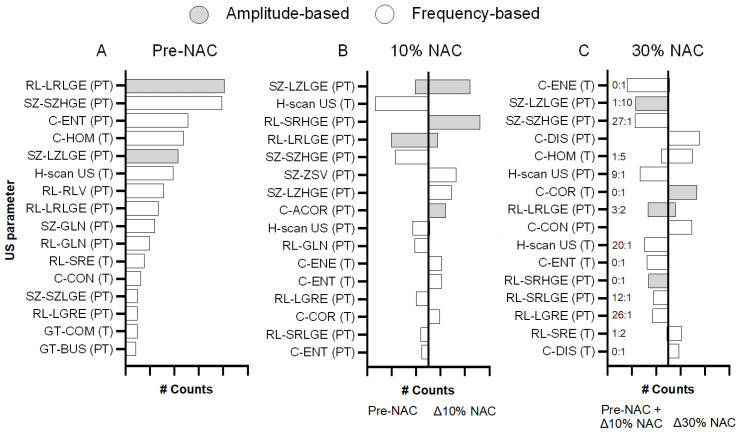
Histogram of the top 16 most frequently selected parameters for optimized performance of the multiparametric US analysis at (**A**) pre-NAC and then after completion of (**B**) 10% and (**C**) 30% of the total NAC regimen. After 30% completion of NAC, the ratio of precursor information from pre-NAC (**A**) and relative measures till 10% NAC completion (**B**) measures is annotated as A:B. These US parameters included texture analysis of either amplitude-based maps (speckle- and B-scan-derived) or frequency-based maps (ACE- and H-scan-derived). Texture parameters include: (1) gray level (GL) co-occurrence (**C**) matrix (GLCM)-based texture assessments, including contrast (CON), correlation (COR), dissimilarity (DIS), entropy (ENT), energy (ENE), and homogeneity (HOM); (2) GL run length (RL) matrix-based features, including GL non-uniformity (GLN), low GL run emphasis (LGRE), long run low GL run emphasis (LRLGE), short run emphasis (SRE), short run high GL emphasis (SRHGE), and short run low gray-level emphasis (SRLGE); (3) size zone (SZ) matrix-based texture features, including small zone high GL emphasis (SZHGE), small zone low GL emphasis (SZLGE), large zone low GL emphasis (LZLGE), large zone high GL emphasis (LZHGE), and zone-size variance (ZSV); and (4) neighborhood gray-tone difference matrix (NGTDM)-based features, including busyness (BUS) and complexity (COM). Mean US parameters were computed for both tumor (T) and peri-tumoral (PT) regions.

The classification performance of the multiparametric US analysis was evaluated in 10 partitions that were iterated 10 times for pCR detection. During the multiparametric US analysis, 78 unique pre-NAC US parameters and 64 unique longitudinal radiomic measurements were selected across 10 partitions. On average, 6.75, 9.29, and 10.43 parameters were selected per partition at pre-NAC and after completion of 10 and 30% of the treatment regimen, respectively. During parameter selection after partial NAC completion, information from the previous assessment was jointly considered with relative changes observed at the current timepoint. Among the selected US parameters, only 41 pre-NAC parameters and 30 parameters describing relative tumor changes from baseline were selected at least five times across all partitions. Notably, US measures from the peri-tumoral region were more commonly selected than those from the tumor area. More precisely, 27 of the 41 frequently selected pre-NAC US parameters described peri-tumoral information. And at early NAC timepoints, 11 (7) and 11 (8) US parameters indicating relative changes in the peri-tumor (tumor) region were selected after completion of 10 and 30% of the NAC regimen, respectively. Among the most frequently occurring US parameters, amplitude-based LRLGE and frequency-based SZHGE from the peri-tumor region were selected more than 80 times at pre-NAC. After 10% completion of the NAC regimen, amplitude-based SRHGE and LZLGE US parameters describing peri-tumoral relative changes were selected more than 70 times for tumor response prediction. And after 30% completion of NAC treatment, frequency-based US parameters DIS (peri-tumor) and amplitude-based COR (tumor) describing relative change from baseline were selected for more than 50% of the total iterations. Figure 7 shows histograms of the 16 most frequently selected US parameters at each NAC timepoint during the multiparametric analysis of the breast cancer data. These US parameter selection analyses highlight the importance of peri-tumoral changes to predict early tumor response as well as the role of frequency-based US radiomic features during pre-NAC separation between both groups.

### 3.4. Multiparametric US Scoring

**Figure 8:**
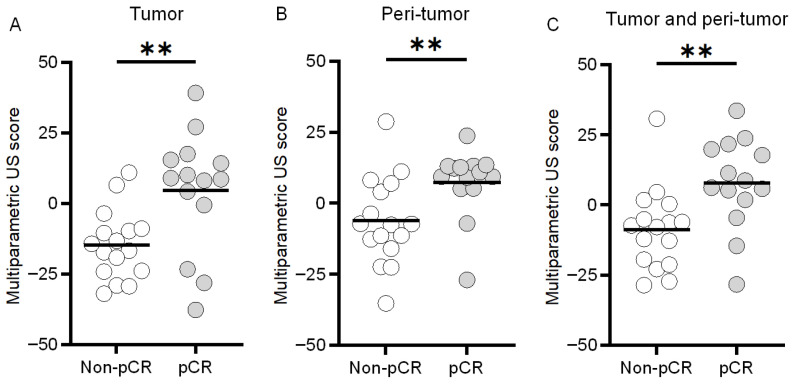
Scatter plots of individual multiparametric US scores at baseline (pre-NAC) for the pCR or non-pCR groups derived from either the (**A**) tumor, (**B**) peri-tumor, or (**C**) combination of both regions (i.e., tumor and peri-tumor). Note that a negative or positive multiparametric US score predicts the likelihood of a non-pCR or pCR tumor response to NAC, respectively. ** indicates *p* < 0.01.

**Figure 9:**
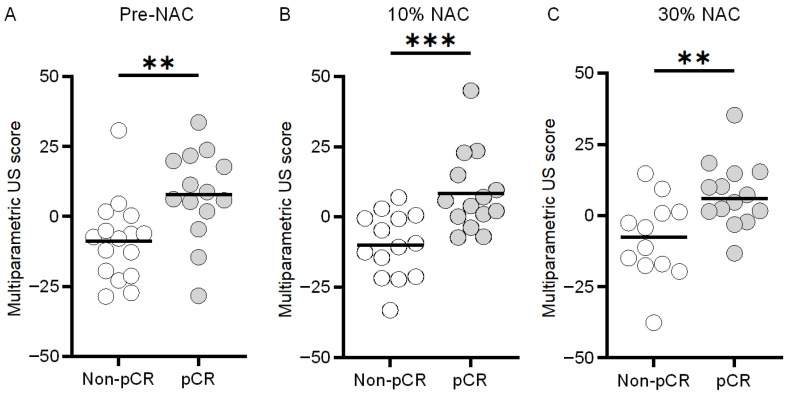
Scatter plots of individual multiparametric US scores for the pCR or non-pCR groups derived at (**A**) baseline (pre-NAC) and after completion of (**B**) 10% and (**C**) 30% of the total NAC regimen. ** indicates *p* < 0.01; *** indicates *p* < 0.001.

With values of 4.6 ± 21.2 and −14.6 ± 12.2, the pre-NAC multiparametric US scores obtained from the tumor region yielded a significant difference between the pCR and non-pCR groups, respectively (*p* < 0.01, Figure 8). While analyzing the peri-tumoral region, multiparametric US scores from analysis of the pCR and non-pCR cases were found to be 7.7 ± 16.1 and −8.7 ± 14.6, respectively (*p* < 0.01), which indicates a subtle increase in the differences between the means of both groups. However, the combination of both tissue regions yielded greater differences between the experimental groups with values of 9.5 ± 10.6 and −13.9 ± 11.0 for the pCR and non-pCR cases, respectively (*p* < 0.01). Notably, while analyzing the multiparametric US scores at early NAC timepoints, after 10% completion of treatment there was a larger difference between both groups (pCR: 8.4 ± 14.3 vs. non-pCR: –10.1 ± 11.6, *p* < 0.001) than pre-NAC assessment (7.7 ± 16.1 vs. –8.7 ± 14.6, *p* < 0.01). After 30% completion of NAC regimen, scores were 7.4 ± 11.6 and –8.2 ± 14.4 (*p* < 0.01) (Figure 9). These results show that the peri-tumoral US measures improve pre-NAC stratification of breast cancer response. Additional integration of relative changes in US measurement after NAC initiation further strengthened this separation.

### 3.5. Multiparametric US Classification

The classification performances using the multiparametric US score indicated that measurements from the breast tumor region prior to the start of NAC yielded a mean AUC of 0.78 with an accuracy of 80.1%, sensitivity of 72.0%, and specificity of 87.1% (see Table 4). Notably, the AUC increased to 0.81 with an accuracy of 76.6%, sensitivity of 85.6%, and specificity of 68.8%, when the analysis only included US measurements from the peritumor region. The integration of baseline information from both tissue regions in the multiparametric US analysis revealed an identical performance (AUC of 0.81). Interestingly, the analysis achieved increased AUC values of 0.86 and 0.83 when incorporating US data obtained from the patients after they completed 10 and 30% of their NAC regimen, respectively (see Table 5). The classification assessment at early treatment timepoints was higher than that found during the baseline assessment alone. Collectively, these results demonstrate that a multiparametric US analysis can help predict a breast cancer response to NAC from baseline measures only, particularly when inclusion of the peri-tumoral information improves the stratification between both the groups. Furthermore, the ability to determine tumor response is improved when early follow-up US measurements are incorporated into the assessment.

## 4. Discussion

This study explored the integration of multiparametric US imaging and radiomic features with machine learning to predict early breast cancer response in patients undergoing NAC. An optimized tumor response scoring system was developed using longitudinal US data from 30 patients. These parameters included spatial heterogeneity data from both the segmented tumor space and a 5 mm wide peri-tumoral region. The objective was to use noninvasive multiparametric US measurements to differentiate pCR from non-pCR in breast cancer patients, with confirmation provided by pathological analysis of surgical specimens.

This study underscores the importance of peri-tumor information for tumor response classification using multiparametric US analyses before NAC initiation. Previous studies using quantitative US imaging have also highlighted the importance of surrounding tissue information for early detection of breast cancer response [52,53]. During analysis of both tumor and peri-tumoral US measurements, Osapoetra et al. described how tumor response classification was improved at baseline (AUC = 0.86) when both tumor and peri-tumor regions were included compared to using each region separately (tumor AUC = 0.79; peri-tumor AUC = 0.82) [44]. These results align with the findings of our study (tumor AUC = 0.78; peri-tumoral AUC = 0.81; combined tumor and peri-tumor regions AUC = 0.81) using texture analysis of B-scan and H-scan US images. The frequency of US parameters selected during the multiparametric US analysis across all partitions annotates the importance of each parameter for tumor response classification. As shown in Figure 7, the peri-tumoral US measurements were the most selected during the multiparametric US analysis before patients started NAC, underscoring the importance of surrounding tissue information for baseline tumor response prediction to treatment.

Previous quantitative US imaging studies also explored repeat measurements to assess early tumor response to NAC. The use of nonlinear SVM has been instrumental in characterizing longitudinal changes in US measurements for tumor response prediction [54]. However, the above-mentioned studies did not include the peri-tumoral analysis while assessing temporal changes. In another study, the role of peri-tumoral regions during longitudinal US measurements for improved tumor response classification was highlighted [55]. More specifically, it was shown that longitudinal peri-tumoral radiomic changes were more prominent than the tumor region in non-pCR patients, whereas for pCRs, the tumor region showed greater changes than the peri-tumor region. This discriminative role of the peri-tumor region after treatment initiation improved stratification between pCRs and non-pCRs. In our study we investigated both tumor and peri-tumor regions during longitudinal assessment. During the feature selection process using the early treatment longitudinal radiomic changes, peri-tumoral information again emerged as the most selected region. Notably, the multiparametric US scores yielded a maximum AUC of 0.86 after only 10% completion of NAC, exceeding the performance observed at baseline (i.e., before starting NAC). These findings underscore the critical role of combining peri-tumoral analysis and early treatment longitudinal radiomics for improved tumor response prediction. Importantly, the observed improvement in predictive performance suggests that this approach has the potential to capture treatment-associated US imaging biomarkers at an early stage of therapy using a relatively simple and robust mathematical framework.

H-scan US frequency imaging can detect differences in tissue scatterers. In this study, a significant positive correlation was observed between pre-NAC H-scan US frequency and Ki-67 levels. Several preclinical studies have shown a strong positive correlation between Ki-67 scores and tumor grade [56]. Prior quantitative US studies have also reported that higher-grade breast cancer patients often exhibit smaller US scatterer sizes [57]. This is relevant to H-scan US imaging that yields higher frequencies for smaller scatterers. In this study, patients who achieved pCR had higher pre-NAC H-scan US frequency values, consistent with the expectation that their tumors contained smaller US scatterers. Supporting evidence from a clinical study of 180 patients further demonstrated that pCR patients tend to have smaller US scatterer sizes before undergoing NAC [52]. Attenuation coefficient estimation used in this study may be impacted due to the presence of the US focus placed in the center of the tumor in this study [58]. Based on findings from prior phantom experiments, a tolerance of up to 7% error in estimation was accepted in this study by limiting post-focal zone analysis to only 0.8 cm.

Research presented in this paper was limited to an analysis of only 30 breast cancer patients. We utilized a nonlinear SVM which requires less data to learn the hyperplane decision. Prior to training the model, the multiparametric US data were reduced using PCA. Dimension reduction helps in mitigating model overfitting [59] and makes the training data sufficient for SVM training [60]. Nonetheless more training points increase the reliability of SVM performance. In this study, we combine multiparametric US data and interpret the decision hyperplane in a 3D plane and display the trajectory over time after treatment initiation, which was used to quantify pCR response. In this pilot study, the increase in AUC score from 0.81 at baseline to 0.86 after NAC initiation highlights the potential of multiparametric US imaging to capture early treatment response-related biomarkers. Since US imaging is cost-effective and widely available, the proposed multiparametric US framework could potentially be incorporated into longitudinal US examinations during patient treatment with NAC to help monitor breast cancer response in a clinical setting and may help identify patients unlikely to achieve pCR at an early stage of therapy, potentially allowing clinicians to consider treatment adaptation strategies. However, studies involving larger patient populations and independent external validation cohorts are necessary to confirm the clinical value and generalizability of the multiparametric US analysis. Tumor response classification should ideally incorporate longitudinal (repeat) measurements and be evaluated within balanced molecular subtype and treatment-stratified groups. Nonetheless, the limited cohort size in the present study did not provide sufficient statistical power for such stratified analyses. In addition, because US provides a cross-sectional view of breast tissue, it may fail to capture the full volumetric complexity of heterogeneous breast cancers. As a result, important variations in the core and peri-tumor region can be missed, leading to incomplete characterization of breast cancers. Future work may explore the use of volumetric H-scan US [61,62] and a multiparametric solution for more comprehensive breast imaging. Notwithstanding, our research results were encouraging and highlighted the use of a multiparametric US solution for early detection of tumor response to NAC.

## 5. Conclusions

H-scan US imaging is a computationally efficient approach to tissue characterization that can be combined with advanced analysis and other modalities to form a multiparametric US approach. It was shown that multiparametric US imaging has the potential to predict the pathological outcome of patients with breast cancer before the start of NAC, and that this prognostic capability can be improved by including early changes in US parameters after treatment initiation. Notably, peri-tumoral US parameters were more frequently selected for optimized tumor response classification. Overall, new clinical technologies like multiparametric US imaging are promising and may enable more personalized and effective patient care strategies.

## Figures and Tables

**Table 1:** Summary of breast patient characteristics recruited for neoadjuvant chemotherapy (NAC).

Patient Characteristics	pCR	Non-pCR
	(*n* = 14)	(*n* = 16)
**Age (years)**		
Median	49	58
Range	27–69	36–68
**Initial tumor size (cm)**		
Median	2.5	2.5
Range	0.9–5.0	1.0–7.2
**Molecular markers**		
Luminal A (ER+/PR+, HER2−)	2/6	4/6
Luminal B (ER+/PR+, HER2+)	4/9	5/9
HER2+	5/10	5/10
**Histological type**	7/14	7/14
IDC	14/29	15/29
IMPC	0/1	1/1

pCR = pathologic complete response, ER+/PR+ = estrogen/progesterone-receptor positive status, HER2+ = human epidermal growth factor receptor 2 positive status, IMPC = invasive micropapillary carcinoma.

**Table 2:** Sensitivity analysis of B-scan ultrasound (US) and H-scan US frequency imaging with residual cancer burden (RCB) scores across varied peri-tumoral tissue width, evaluated using correlation analyses (*r*, *p*-values).

			Surrounding Peri-Tumor Tissue (Width)			
			3 mm	5 mm	7 mm	10 mm
**H-scan US**		Pre-NAC	0.41, 0.03	0.42, 0.03	0.40, 0.04	0.39, 0.05
		10% NAC	0.21, 0.30	0.19, 0.34	0.31, 0.12	0.25, 0.20
		30% NAC	0.28, 0.15	0.30, 0.13	0.29, 0.15	0.29, 0.15
**B-scan US**		Pre-NAC	0.08, 0.67	0.12, 0.57	0.15, 0.45	0.14, 0.50
		10% NAC	0.23, 0.25	0.30, 0.13	0.34, 0.08	0.35, 0.08
		30% NAC	0.24, 0.24	0.22, 0.28	0.16, 0.42	0.11, 0.57

**Table 3:** Summary of mean attenuation coefficient estimates α (dB/cm/MHz) for patients achieving pCR and non-pCR at baseline (pre-NAC) and after completion of 10 and 30% of the NAC treatment regimen.

	Pre-NAC	10% NAC	30% NAC
		**Tumor**	
pCR	−0.35 ± 0.21	−0.36 ± 0.23	−0.45 ± 0.24
Non-pCR	−0.54 ± 0.25, *p* = 0.07	−0.52 ± 0.20, *p* = 0.07	−0.61 ± 0.21, *p* = 0.09
		**Peri-tumor**	
pCR	−0.34 ± 0.20	−0.36 ± 0.19	−0.40 ± 0.21
Non-pCR	−0.51 ± 0.22, *p* = 0.08	−0.46 ± 0.17, *p* = 0.17	−0.52 ± 0.13, *p* = 0.08

**Table 4:** Performance of optimal multiparametric US combinations using baseline (pre-NAC) data collected from the tumor, peri-tumor, or combination of both tissue regions.

AUC	Accuracy (%)	Sensitivity (%)	Specificity (%)	*p*-Value
[95% CI]	[95% CI]	[95% CI]	[95% CI]	
		**Tumor**		
0.78	80.1	72.0	87.1	<0.01
[0.59, 0.98]	[65.8, 94.3]	[48.7, 95.9]	[70.7, 100]	
		**Peri-tumor**		
0.81	76.6	85.6	68.8	<0.01
[0.63, 0.98]	[61.6, 91.6]	[67.0, 91.6]	[46.4, 91.3]	
	**Tumor + peri-tumor**			
0.81	76.6	78.4	75.3	<0.01
[0.63, 0.99]	[60.8, 92.5]	[56.0, 100]	[53.8, 96.7]	

**Table 5:** Performance of the optimal multiparametric US combinations data collected at baseline (pre-NAC) and early timepoints after completion of 10 and 30% of the total NAC regimen.

**AUC**	**Accuracy (%)**	**Sensitivity (%)**	**Specificity (%)**	***p*-Value**
**[95% CI]**	**[95% CI]**	**[95% CI]**	**[95% CI]**	
		**Pre-NAC**		
0.81	76.6	78.4	75.3	<0.01
[0.63, 0.99]	[60.8, 92.5]	[56.0, 100]	[53.8, 96.7]	
		**10% NAC**		
0.86	78.9	79.2	78.6	<0.001
[0.73, 1.0]	[64.0, 93.6]	[57.8, 100]	[56.7, 100]	
	**30% NAC**			
0.83	73.2	78.5	67.3	<0.01
[0.65, 1.0]	[55.6, 91.0]	[56.4, 100]	[39.5, 95.1]	

## Data Availability

Data is available upon reasonable request.
